# Innovative Uses of Condom Uterine Balloon Tamponade for Postpartum Hemorrhage in India and Tanzania

**DOI:** 10.1155/2018/4952048

**Published:** 2018-06-03

**Authors:** Jennifer Makin, Daniela I. Suarez-Rebling, Poonam Varma Shivkumar, Vincent Tarimo, Thomas F. Burke

**Affiliations:** ^1^Massachusetts General Hospital Division of Global Health and Human Rights, Boston, MA, USA; ^2^Harvard T.H. Chan School of Public Health, Boston, MA, USA; ^3^SUNY Upstate Medical University Department of Obstetrics and Gynecology, Syracuse, NY, USA; ^4^Mahatma Gandhi Institute of Medical Sciences, Sevagram, India; ^5^Muhimbili National Hospital, Dar es Salaam, Tanzania; ^6^Harvard Medical School, Boston, MA, USA

## Abstract

**Background:**

Postpartum hemorrhage is the most common cause of maternal deaths worldwide, the majority of which occur in low-resource settings. Uterine balloon tamponade (UBT) is an effective method of addressing uncontrolled postpartum hemorrhage (PPH) from uterine atony; however, UBT devices are often not affordable. We report on three novel uses of an ultra-low-cost condom uterine balloon tamponade (ESM-UBT) device.

**Cases:**

ESM-UBT devices were used in innovative ways to arrest severe uncontrolled pregnancy-related hemorrhage among three women in India and Tanzania. The first had sustained deep vaginal lacerations, the second a cervical pregnancy, and the third a complete molar pregnancy.

**Conclusion:**

The ESM-UBT device may be useful for control of obstetric hemorrhage caused by complex vaginal tears as well as cervical and molar pregnancies.

## 1. Introduction

Postpartum hemorrhage (PPH) accounts for approximately 127,000 deaths worldwide annually, 99% of which occur in low-resource settings [1]. Survival of women with severe PPH is dependent on high-quality timely care. The initial step in caring for women with PPH is to identify the source of blood loss. Uterine atony, the most common cause of PPH, should be managed in a deliberate fashion beginning with fundal massage, bladder drainage, and administration of uterotonics, followed by aortic compression and rapid placement of a uterine balloon tamponade (UBT) device should hemorrhage continue unabated [1].

Tamponade devices, for management of bladder and esophageal hemorrhage, were first described over 50 years ago [2, 3]. In 1983, Goldrath reported on the use of Foley catheters as tamponade devices to arrest postpartum hemorrhage [4]. Since then, commercial UBT devices have become available for use in atonic postpartum hemorrhage; however they are often unaffordable in resource limited settings. Over the past eight years the authors have designed, implemented, refined, and studied [5, 6] an evidence-based package called Every Second Matters for Mothers-UBT (ESM-UBT). The ESM-UBT is a simple innovation that consists of a condom fastened to a 24 French Foley catheter by string. While a clean glove had been considered as a low-cost solution, the condom was pursued because it is a low pressure system which can accommodate a high volume and conforms to the space it is inflated within.

To use the ESM-UBT, clean water is used to inflate the condom through the catheter using a syringe and a one-way valve. The condom balloon is inflated until the bleeding is arrested. The ESM-UBT device is safe and extremely effective in saving women's lives, especially when placed before the advanced stages of shock [6]. The authors have supported implementation of the ESM-UBT package across several low-resource countries including South Sudan, Kenya, Sierra Leone, Senegal, Zambia, Ghana, Nepal, India, Uganda, Honduras, Peru, Tanzania, and Cote' Ivoire.

In this article, the authors report on three innovative uses of ESM-UBT devices for severe PPH originating from sources other than atonic uterus. These cases came to the authors' attention over the course of their collaborative efforts on implementation of the ESM-UBT package in India and Tanzania.

## 2. Cases

### 2.1. Case 1

A 26-year-old primigravida at 39-week gestation by date of last menstrual period, presented to the Muhimbili National Referral Hospital, Tanzania, in active labor. She had no significant past medical history and an uncomplicated antenatal course. Shortly after midnight she had a precipitous vaginal delivery. After delivery, the woman began hemorrhaging profusely. Her uterus was vigorously massaged, ten international units (IU) of intravenous oxytocin were administered, and the placenta manually removed. Despite the interventions, the patient continued to bleed and lost consciousness. On pelvic examination, the uterus was well contracted, bleeding superior to her cervix was minimal, and second degree bilateral vaginal sulcal lacerations were identified. Fluid resuscitation was initiated, and the patient was emergently taken to the operating theatre by the in-house Obstetrician and Gynecology resident for examination under anesthesia and repair of her vaginal lacerations. Despite attempts at suture repair, hemorrhage from the laceration sites continued. The senior Obstetrician and Gynecology consultant was also unsuccessful in gaining control of the hemorrhage and therefore placed an ESM-UBT device into her vagina. The ESM-UBT device was inflated with 300 cc of water and secured with vaginal packing. Hemorrhage ceased and antibiotics, intravenous fluids, and blood products were administered. The ESM-UBT device was removed after 48 hours, no further repair was necessary, and bleeding did not recur. The woman was discharged home on postpartum day two and at her six-week postpartum visit had fully recovered.

### 2.2. Case 2

A 22-year-old gravida two para one woman, six-week pregnant by dating from her last menstrual period, presented to a private maternal health facility for termination of her pregnancy via dilation and suction curettage. Upon attempted cervical dilation the woman began hemorrhaging profusely. She was referred by ambulance to the Mahatma Gandhi Institute for Medical Sciences where she presented extremely pale, weak, and in shock with vital signs including a blood pressure of 80/30 mm/Hg and heart rate of 145 bpm. Outside imaging that was brought with the patient, when reviewed at the referral facility, clearly identified a cervical pregnancy. The patient was emergently taken to the operating theatre where resuscitation was initiated, and a rapid pelvic exam was performed. Examination revealed severe bleeding and a swollen cervix ballooned with clots. An ESM-UBT device was intentionally placed into the cervix, inflated with 200 cc of water which formed a dumbbell, filling the cervix and ballooning into the lower uterine segment and vagina. Vaginal packing was used to secure the uterine balloon's position. The woman was transferred to the intensive care unit where she received isotonic fluids and two units of blood. The ESM-UBT device was removed after 24 hours without any resumption of bleeding. There were no complications on six-week follow-up.

### 2.3. Case 3

A 27-year-old gravida three para two woman, 23-week pregnant by date from her last menstrual period, presented to the Government Medical College Nagpur National Hospital, India, with vaginal bleeding. She had no significant past medical history. On physical examination, her fundal height was consistent with a 32-week uterus, there was moderate vaginal bleeding, and her cervix was closed. An ultrasound examination was remarkable for absence of a fetus and a solid collection of echoes with numerous anechoic spaces consistent with a molar pregnancy. The patient was taken to the operating theatre for cervical dilation and evacuation under general anesthesia. Following evacuation, the uterus was atonic and hemorrhage ensued with an estimated blood loss of greater than two liters. Bimanual uterine massage was performed, the bladder was drained, fluid resuscitation was initiated, and oxytocin (20 IU) and ergometrine (0.4 mg) were administered intravenously. Despite these interventions, profuse bleeding continued, and her vital signs deteriorated to a blood pressure of 84/60 and heart rate of 120. An ESM-UBT device was inserted into the uterus, inflated with 300 cc of water, and hemorrhage was immediately arrested. Her condition stabilized, and she was transfused blood. The ESM-UBT device was removed after 24 hours with no bleeding recurrence and the woman was discharged home two days thereafter. Pathology results confirmed a complete molar pregnancy. There were no complications on subsequent two- and six-week follow-up visits and she remains within their system for clinical monitoring throughout the year.

## 3. Discussion

Uncontrolled postpartum hemorrhage is the most common cause of maternal death and disability worldwide. Although uterine atony following a spontaneous vaginal delivery is the most frequent reason for PPH, other pregnancy-related emergent conditions may also lead to rapid blood loss [1]. In these three cases, we report on innovative uses of a condom UBT for the management of severe hemorrhage from complex vaginal lacerations, a cervical pregnancy, and a molar pregnancy.

Previous reports have described the use of balloon tamponade to arrest uncontrolled hemorrhage from vaginal lacerations [7], cervical pregnancies [8], and molar pregnancies [9]. However, these cases were managed in well-resourced facilities using proprietary balloon tamponade devices, often in combination with other advanced interventions such as cerclage placement and methotrexate administration. 18 fr Foleys have been used successfully for tamponade of cervical pregnancies in the past; however, in this case the condom system provided the volume required for adequate tamponade. The three cases described in this report represent innovative applications of the ultra-low-cost ESM-UBT device in limited resource settings where advanced surgical and technology interventions are not available.

In summary, these three cases illustrate that the ESM-UBT device may be applied in creative ways and there may be emergent conditions other than hemorrhage from atonic uterus where an ESM-UBT device can help save lives.

## References

[1] World Health Organization Reproductive Health. Managing Complications in Pregnancy and Childbirth: A Guide for Midwives and Doctors.

[2] Helmstein K. (1972). Treatment of bladder carcinoma by a hydrostatic pressure technique report on 43 cases. *British Journal of Urology*.

[3] Sengstaken R. W., Blakemore A. H. (1950). Balloon tamponade for the control of hemorrhage from esophageal varices. *Annals of Surgery*.

[4] Goldrath M. H. (1983). Uterine tamponade for the control of acute uterine bleeding. *American Journal of Obstetrics & Gynecology*.

[5] Burke T. F., Ahn R., Nelson B. D. (2016). A postpartum haemorrhage package with condom uterine balloon tamponade: a prospective multi-centre case series in Kenya, Sierra Leone, Senegal, and Nepal. *BJOG: An International Journal of Obstetrics & Gynaecology*.

[6] Burke T. F., Danso-Bamfo S., Guha M., Oguttu M., Tarimo V., Nelson B. D. (2017). Shock progression and survival after use of a condom uterine balloon tamponade package in women with uncontrolled postpartum hemorrhage. *International Journal of Gynecology and Obstetrics*.

[7] Tattersall M., Braithwaite W. (2007). Balloon tamponade for vaginal lacerations causing severe postpartum haemorrhage. *BJOG: An International Journal of Obstetrics & Gynaecology*.

[8] Bakour S. H., Thompson P. K., Khan K. S. (2005). Successful conservative management of cervical ectopic pregnancy with combination of methotrexate, mifepristone, surgical evacuation and tamponade using a double balloon three-way catheter. *Journal of Obstetrics & Gynaecology*.

[9] Kolomeyevskaya N. V., Tanyi J. L., Coleman N. M., Beasley A. D., Miller H. J., Anderson M. L. (2009). Balloon tamponade of hemorrhage after uterine curettage for gestational trophoblastic disease. *Obstetrics & Gynecology*.

